# Evaluation of the Protective Effect of a Nanoscale Deep Penetration Sealer in Improving Chloride Erosion Resistance of Concrete

**DOI:** 10.3390/ma17235755

**Published:** 2024-11-25

**Authors:** Yan Liu, Xiaoli Peng, Jia Hui, Peng Zhang, Zhiqian Zhang

**Affiliations:** 1 School of Architectural Engineering and Surveying & Mapping Engineering, Jiangxi University of Science and Technology, Ganzhou 341000, China; 6720221273@mail.jxust.edu.cn; 2 School of Civil Engineering and Architecture, Jiangxi College of Applied Technology, Ganzhou 341000, China; 13576691483@163.com; 3 China Academy of Transportation Science, Beijing 100029, China; huijiabuct@126.com; 4 Yichun Lithium New Energy Industry Research Institute, Jiangxi University of Science and Technology, Yichun 336023, China; 9120180103@jxust.edu.cn

**Keywords:** chloride diffusion law, durability, concrete surface treatment, nanoscale deep penetration sealer, prediction model

## Abstract

In this study, the protective effect of a Nanoscale Deep Penetration Sealer (NDPS) in improving the chloride erosion resistance of concrete was evaluated and the influence of water–cement ratio (*w*/*c*) and the NDPS spray volume on the protective effect was explored, in order to gain a deeper insight into the effect of NDPS on the durability of concrete in chloride environments. The thickness of the protective layer formed by NDPS within the concrete was determined and the effectiveness of this protective layer was verified. Based on the determination of the ability of NDPS to form a protective layer in concrete, the diffusion laws of chloride in concrete at different *w*/*c* and NDPS spray volumes were investigated, and a prediction model was established. The results show that NDPS forms a 2–3 cm protective layer in concrete to resist chloride penetration, which is nearly as thick as the concrete cover. The protective layer weakens the capillary absorption of concrete and prevents the penetration of aggressive substances into the concrete. NDPS significantly improves the chloride erosion resistance of concrete. The chloride diffusion coefficient of concrete with a *w*/*c* ratio of 0.6 was reduced by approximately 35% after being sprayed with 1000 mL/m^2^ of NDPS, and the protective effect strengthens with increasing spray volume at a fixed *w*/*c* and weakens with decreasing *w*/*c* at a fixed NDPS spray volume. The proposed predictive model is the basis for predicting the diffusion of chloride in concrete with NDPS protection in practical engineering applications and provides a guide for the application of NDPS in practical engineering.

## 1. Introduction

On account of its low cost, abundant raw materials, and high compressive strength, concrete is widely used as an artificial building material. However, the pore structures in concrete that are connected to the external environment provide a channel for the transport of aggressive substances [1]. This is extremely fatal to reinforced concrete structures in marine environments [2]. In the marine environment, chloride is rapidly transported to concrete by diffusion, convection, and electromigration. As chloride concentration rises to a critical level, the protective passivation film of steel in concrete is depassivated, resulting in corrosion of the steel [3,4].

**Figure 1:**
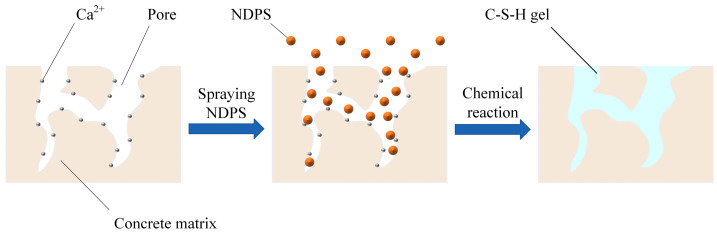
Schematic diagram of the mechanism of action of NDPS.

The application of surface treatments to concrete represents an effective method of preventing the ingress of aggressive substances [5]. The method is applicable to both existing and pending concrete structures, distinguishing it from other protective methods. According to the active mechanism, surface treatments are grouped into polymer coating, hydrophobic impregnation, and pore-blocking surface treatment [6]. Polymer coating represented by traditional epoxy resins and polyurethane is usually construction complex, low efficiency, poor environmental protection, prone to aging, and low economic benefit [7]. Silanes and siloxanes are hydrophobic impregnations that provide protection by penetrating concrete to form a hydrophobic film. This is costly and less weather resistant [8]. More seriously, its protection fails when the concrete is cracked. Pore-blocking surface treatment provides better weather resistance and durability than polymer coating and hydrophobic impregnation [9]. The treatment provides permanent protection for concrete by blocking microscopic pores with a gel, which is formed by a chemical reaction between it and the concrete matrix [10,11,12]. NDPS is a pore-blocking surface treatment that has attracted attention in recent years. The NDPS is developed on the basis of the Deep Penetration Sealer (DPS). The NDPS is made of alkali silicate as the base material with the addition of active substances and catalysts by nanotechnology. The action mechanism of NDPS is shown in Figure 1. The NDPS blocks the pores with calcium silicate-hydrated colloid (C-S-H), which is produced by a chemical reaction with free Ca^2+^ in the pores [13]. The transmission pathways for harmful substances are broken as the pores are blocked. This leads to challenges in the penetration and transport of harmful substances within concrete. In this way, the durability of the concrete was fundamentally improved. NDPS is superior to DPS in penetration speed, penetration depth, and reaction speed [14]. In addition, it has the advantages of convenient construction, non-toxicity, powerful weathering resistance, and long-lasting protective effects. In summary, NDPS has great potential in engineering applications.

In previous studies, Coulombic flux and rapid chloride migration (RCM) methods are the primary methods used by scholars to evaluate the chloride erosion resistance of concrete with surface treatment [15,16,17,18]. Chen et al. [19] measured the electrical flux of concrete under pore-blocking surface treatment by the coulomb electrical flux method and concluded that the pore-blocking surface treatment effectively improves the durability of concrete. Zhang et al. [20] investigated the chloride transport in cracked concrete with pore-blocking surface treatment by the Coulombic flux method and concluded that cracked concrete with pore-blocking surface treatment exhibits self-healing properties and similar chloride transport as uncracked concrete. Xiao et al. [21] determined the chloride diffusion coefficients of concrete sprayed with NDPS at different ages by the RCM method and concluded that spraying NDPS was effective in reducing the chloride diffusion coefficient of concrete. The reduction rate exhibited a decreasing trend with the concrete age while spraying NDPS. Tan et al. [22] employed the RCM method to ascertain the chloride diffusion coefficients of engineered cementitious composites (ECC) with varying qualities of pore-blocking surface treatment material. The researchers discovered that the pore-blocking surface treatment material with an optimal quality employed was able to maximize the chloride erosion resistance of ECC. The above studies demonstrate that pore-blocking surface treatments effectively improve the chloride penetration resistance of concrete. Nevertheless, these studies lacked both quantitative analyses of the influences of *w*/*c* and protective material dosage on the protective performance of pore-blocking surface treatment, and further development of predictive models for evaluating the service life of concrete with pore-blocking surface treatment. These research gaps should be thoroughly explored to facilitate the better application of NDPS in practical engineering. Furthermore, previous studies showed that the accuracy of the Coulombic flux method to investigate the chloride penetration resistance of concrete is poor [23]. More critically, it is difficult to use the data, which is obtained through the Coulombic flux method, to directly establish the necessary predictive models for protection life of pore-blocking surface treatments. The more accurate RCM method also suffers from this flaw [24]. Compared with the RCM method and the Coulombic electric flux, the natural diffusion method is the closest to the chloride migration in concrete of practical engineering and provides a more direct reflection of the chloride diffusion in concrete [25,26]. The method facilitates the analysis of chloride transport patterns in concrete with NDPS protection and the development of a model for predicting chloride diffusion in this case.

In the present study, the focus is on accurately evaluating the protective effect of NDPS in improving the chloride erosion resistance of concrete and revealing the influences of the NDPS spray volume and *w*/*c* on the protective effect. The ability of NDPS to form a protective layer in concrete was the basis of this study. Firstly, the penetration depth of NDPS in concrete was measured by X-ray energy dispersive spectroscopy (EDS) analysis to determine the thickness of the protective layer. Subsequently, the capillary absorption coefficient of the concrete treated with NDPS was measured by the capillary absorption test, and the variation in absorption capacity was analyzed to verify the effectiveness of the protective layer. After that, the chloride concentration distribution of concrete with NDPS protection was determined by an indoor chloride natural diffusion test, and the influences of the NDPS spray volume and *w*/*c* on the protective effect of NDPS were quantitatively analyzed. Finally, a model for predicting chloride diffusion in concrete protected by NDPS was developed and validated to guide engineering practice.

## 2. Materials and Methods

### 2.1. Materials and Specimen Preparation

**Figure 2:**
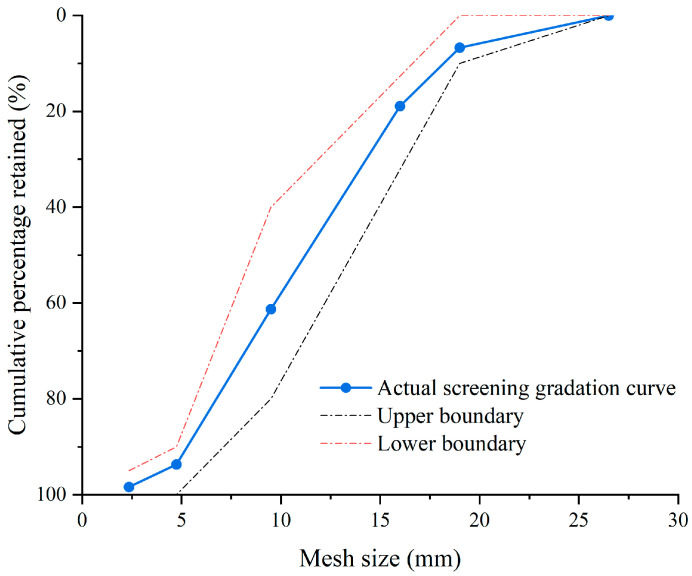
Screening gradation curves for coarse aggregate.

The concrete specimens for the tests were prepared from Ordinary Portland cement (P.O. 42.5), water, fine and coarse aggregates. The basic information of cement is shown in Table 1. The fine aggregate was well-graded river sand with a fineness modulus of 2.8. The coarse aggregate was continuously graded crushed stone with a grain size of 5 to 20 mm, as shown in Figure 2. The distilled water was used during specimen pouring, curing, and experimentation aim at avoiding the influence of chloride in the raw materials of the concrete mixture on the follow-up tests. NDPS produced by Yishengyuan Environmental Protection Engineering Ltd. of Beijing, China was used for the surface treatment of the specimens.

Concrete with a higher water–cement ratio is more susceptible to chloride erosion. Therefore, concrete specimens with water–cement ratios of 0.4, 0.5, and 0.6 were prepared for testing. The proportion of concrete is shown in Table 2. In the treatment of concrete with NDPS, spray volumes of 300 mL/m^2^ and 500 mL/m^2^ were selected according to the recommendations of the manufacturer. In addition, a spray volume of 1000 mL/m^2^ was added to further investigate the NDPS protective effect. The test case design is shown in Table 3.

Concrete specimens were prepared in accordance with the JGJ55-2011 [27]. NDPS was sprayed while the specimens were cured in an environment with a temperature of (23 ± 2) °C and a humidity higher than 90% to an age of 7 d. Before the NDPS was sprayed, the specimen surface was sandpapered. After that, one surface was selected as the protected surface to be sprayed with NDPS. To ensure that the NDPS only penetrated the protection surface, tape was used to form a weir around the protection surface. Furthermore, the specimens were sprayed with NDPS several times for each case to ensure NDPS adequately penetrated into the specimens. After the specimens were sprayed with NDPS, the specimens with dry surfaces continued to cure until the age of 28 d, then experiments were carried out.

### 2.2. EDS Analysis

EDS analysis is fundamental to the study of the protective properties of NDPS to analyze the permeability of NDPS and to examine the ability of NDPS to form a protective layer within the concrete. The NDPS, mainly composed of sodium and potassium silicate, leads to increased sodium and potassium content in the penetration area when it penetrates the concrete. The distribution of Na and K elements in the specimen can be determined by EDS analysis and the depth of the drop in Na and K elements content can be taken as the NDPS protective layer thickness. The specimen groupings for EDS analysis are shown in Table 4.

**Figure 3:**
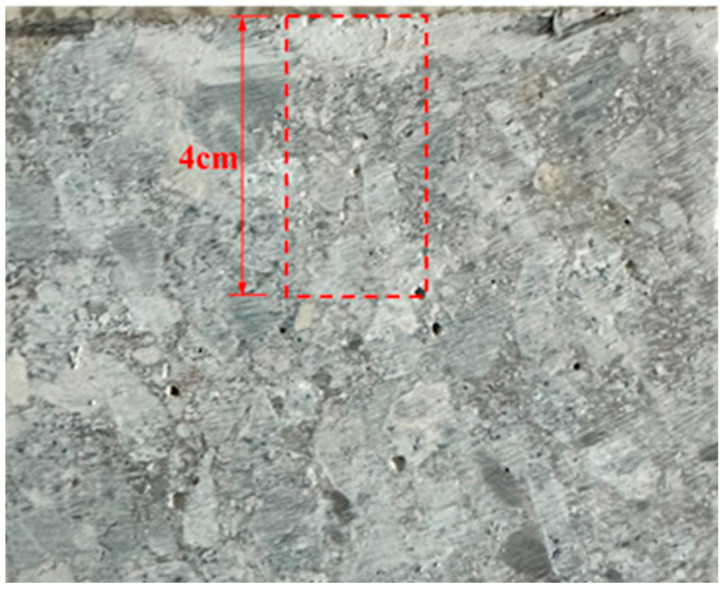
EDS analysis line scan area.

The energy dispersive spectrometer of the MLA650F field emission scanning electron microscope was used for EDS analysis. Before EDS analysis, the specimens were sliced perpendicular to the protected surface to obtain thin slices for scanning within 4 cm from the protected surface. The specimen was sliced in the area shown in the Figure 3. During the process of EDS analysis, the spectrometer was set up with a magnification of 32x for linear scanning of slices, each measuring 5 mm in length, to obtain the distribution of Na and K elements content in the specimens. And the line scans avoid areas with large amounts of coarse aggregate in the specimen slices to guarantee the precision of the results.

### 2.3. The Capillary Water Absorption Test

As the pores of concrete are blocked after being sprayed with NDPS, it inevitably leads to variations in the capillary water absorption coefficient [11,28]. Therefore, the degree of pore blockage in concrete was reflected by variations in the capillary absorption coefficient of the specimens.

**Figure 4:**
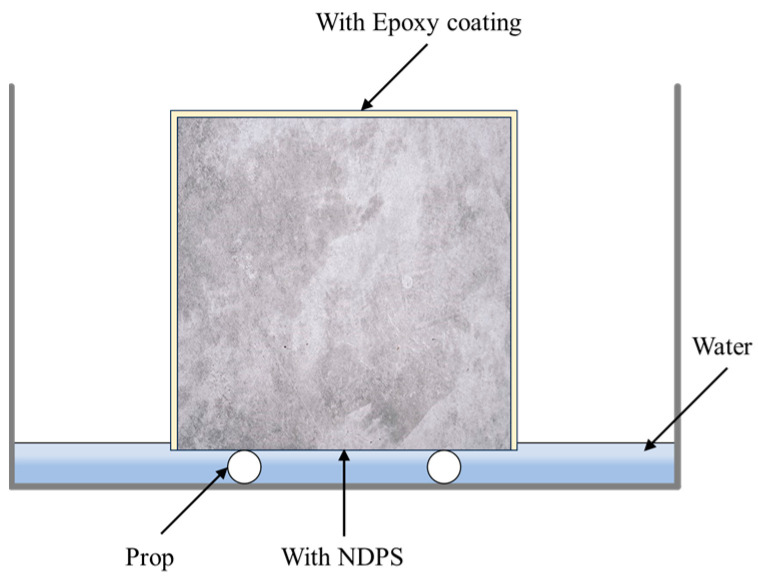
EDS analysis line scan area.

Test conducted in accordance with ASTM C1585 [29]. Before testing, the specimen was dried to a constant weight and sealed with epoxy except for the protected surface. The equipment for the test is shown in Figure 4. During the test, the specimen was placed on prop and the water surface was approximately 3 mm above the protected surface of the specimen. The water absorption of the specimens for the test duration of 24 h was measured, and the test results were averaged over three specimens. Finally, the capillary water absorption coefficient of the specimen was determined by Equation (1):

(1) $$A\mathrm{cap}=\frac{Vw}{Ac\cdot \sqrt{t}}$$

where *A*_cap_ is the capillary water absorption coefficient (mm/h^1/2^), *V*_w_ is the volume of water absorbed (mm^3^), *A*_c_ is the cross-sectional area of the specimen in contact with water (mm^2^), *t* is the water absorption time (h).

### 2.4. The Indoor Natural Diffusion Test

**Figure 5:**
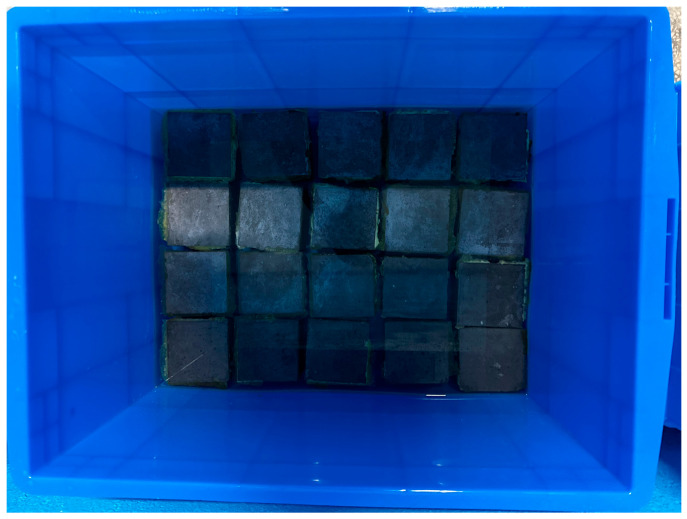
Schematic diagram of exposure environment.

In the test, the 3.5 wt.% NaCl solution with chloride content close to that of actual seawater was used as the immersion solution and the specimens were submerged to simulate concrete underwater in an ocean environment [30,31]. Before immersion, the specimen surfaces were sealed with epoxy except for the protected surface to ensure one-dimensional diffusion of the chloride on the protected surface. During immersion, the specimens were placed in the immersion chamber with the protected surface facing upwards, and the NaCl solution level was approximately 3 cm above the protected surface of the specimen, as shown in Figure 5. According to Table 3, the specimens were immersed for 30, 60, 90, 120 and 150 d, and the solution was changed every 7 d during immersion.

**Figure 6:**
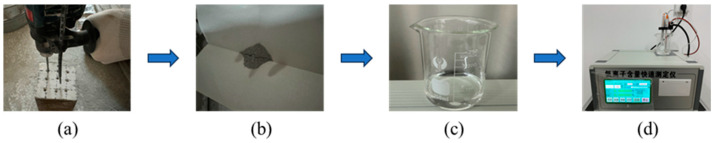
Samples and chloride concentration measurement process: (**a**) drilling of specimens; (**b**) sieved powder sample; (**c**) finished filtrate; (**d**) chloride concentration detection.

The distribution of chloride concentration within the specimen was measured after the specimen achieved the desired immersion time, and the measurement process is shown in Figure 6. Firstly, the drill was used to obtain powder samples at depths of 0–5, 5–10, 10–15, 15–20, 20–25, 25–30, 30–35 and 35–40 mm perpendicular to the protected surface of the specimen. The average depths of 2.5, 7.5, 12.5, 17.5, 22.5, 27.5, 32.5, and 37.5 mm were used to represent the range of depths for each powder sample to facilitate data processing. Furthermore, the holes were meticulously cleaned with a brush during the drilling of the powder samples, and larger particles were separated from the sample using a sieve with a 0.15 mm pore size. After that, the powdered specimens were dried according to JTJ 270-98 [32]. Before measuring the chloride concentration in the powder sample, dissolve 2 g of the powder sample in 40 mL of distilled water. Then it is heated for 5 min and then cooled for 24 h to ensure that the chloride is adequately dissolved in distilled water. Finally, the solution was filtered to obtain a finished filtrate for the test. According to the JGJ/T322-2013 [33], the chloride concentration in the powder samples was obtained by measuring the finished filtrate with DCCL-816 automatic potentiometric titrator. The duration of the test was 1 min for each powder sample.

### 2.5. Model Establishment and Validation Process

The chloride transport prediction model for NDPS-sprayed concrete was established by modifying the existing model for ordinary concrete based on the results of the indoor natural diffusion test. Prior to the model establishment, the shortcomings of commonly used models describing chloride diffusion in concrete were analyzed and relevant assumptions were made for the subsequent work. Subsequently, the time dependence of the key parameters in the model, along with the influence of NDPS spray volume and *w*/*c* on these parameters, was analyzed. After that, a suitable function was selected to perform a regression analysis of the relevant parameters to establish the new model. The accuracy of the fitted model was ensured by the controlling coefficient of determination R^2^ to remain at a high value during this regression analysis. Finally, the accuracy and stability of the new model were verified by error margin analysis and sensitivity analysis.

## 3. Test Results and Discussion

### 3.1. The Penetration Depth of NDPS

**Figure 7:**
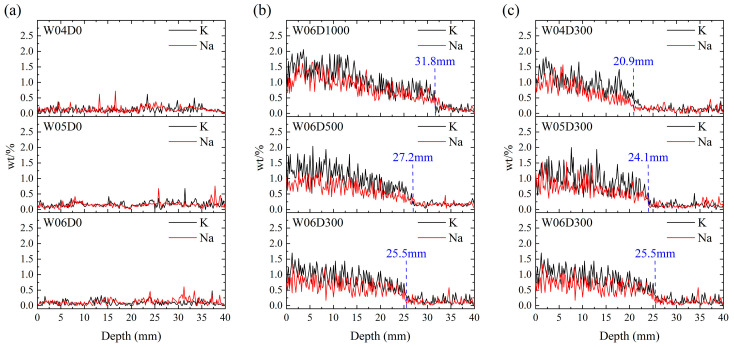
Content variation in K, Na in the specimens: (**a**) Control group; (**b**) Influence of NDPS spray volume; (**c**) Influence of *w*/*c*.

The results of the EDS analysis are presented in Figure 7. As shown in Figure 7a, the Na and K elements content at each depth in the specimens not sprayed with NDPS is close to 0 and varies more smoothly with depth. However, the Na and K elements content in the specimens sprayed with NDPS decreases gradually with depth, and drops abruptly at a certain depth, as shown in Figure 7b,c. The above phenomenon supports the rationale of considering the drop depths of Na and K elements content as the penetration depth of NDPS in concrete.

From Figure 7, it can be concluded that NDPS is highly permeable and can penetrate concrete to a depth of approximately 2–3 cm. The thickness of the protective layer provided by NDPS is similar to the thickness of the concrete cover. Moreover, the permeability of NDPS improves with the increase in the NDPS spray volume and *w*/*c*. For the specimens sprayed with 300 mL/m^2^ NDPS, the penetration depth of NDPS decreased from 25.5 mm to 20.9 mm as the *w*/*c* decreased from 0.6 to 0.4. For the specimens with *w*/*c* of 0.6, the penetration depth of NDPS increased from 25.5 mm to 31.8 mm as the NDPS spray volume increased from 300 mL/m^2^ to 1000 mL/m^2^. In summary, although the difference in material suppliers resulted in the NDPS penetration depth in concrete in this study not achieving the 6 cm reported by Xiao et al. [21], it still substantially exceeded the penetration depth of the DPS.

### 3.2. The Absorption Capacity of Concrete

**Figure 8:**
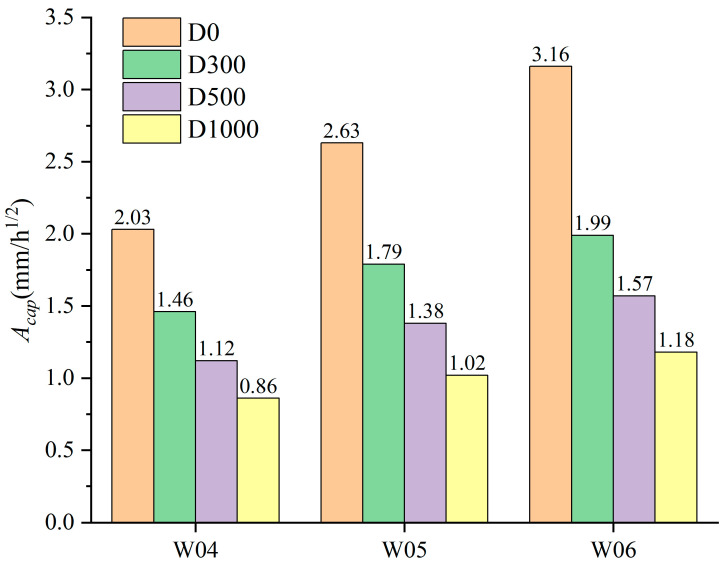
Comparison of capillary absorption coefficient of specimens.

A comparison of capillary water absorption coefficients of specimens is presented in Figure 8. It can be observed that spraying NDPS significantly weakens the capillary water absorption properties of concrete. With increasing NDPS spray volume, the capillary absorption coefficient of concrete with the same *w*/*c* tended to decrease, and the decreasing trend slowed. For example, the capillary water absorption coefficients of the specimens with *w*/*c* of 0.4 were reduced by 28%, 45% and 58% when the NDPS spray volume was increased from 0 mL/m^2^ to 300 mL/m^2^, 500 mL/m^2^ and 1000 mL/m^2^, respectively. With decreasing *w*/*c*, the capillary absorption coefficient of concrete with the same NDPS spray volume tends to decrease and the decreasing trend rises. When the NDPS spray volume was increased from 0 mL/m^2^ to 1000 mL/m^2^, the capillary absorption coefficients of the specimens with *w*/*c* of 0.4, 0.5, and 0.6 were reduced by approximately 57%, 61%, and 63%, respectively.

The decline in the capillary absorption coefficient of concrete is associated with variations in the pore structure. After spraying with NDPS, the active substances within NDPS react to block the pores within the penetration range of NDPS, resulting in a reduction in permeable channels and a weakening of the absorption capacity of concrete. The above phenomenon demonstrates the ability of NDPS to form an effective protective layer in concrete. This demonstrates that the protective layer formed by NDPS can effectively function to hinder the transmission of harmful substances carried by water within the concrete.

### 3.3. Distribution Law of Chloride Content

**Figure 9:**
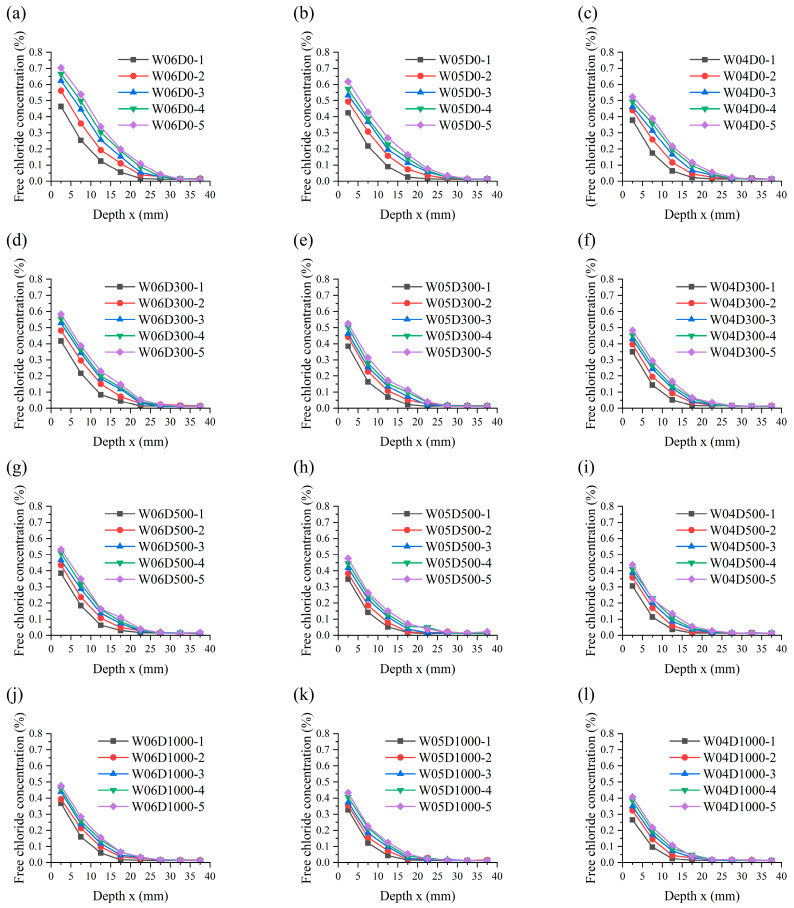
The experimental chloride profiles: (**a**) Case W06D0; (**b**) Case W05D0; (**c**) Case W04D0; (**d**) Case W06D300; (**e**) Case W05D300; (**f**) Case W04D300; (**g**) Case W06D500; (**h**) Case W05D500; (**i**) Case W04D500; (**j**) Case W06D1000; (**k**) Case W05D1000; (**l**) Case W04D1000.

Figure 9 displays the profiles of chloride concentration distribution in the specimens. The figure reveals the common laws of chloride transport in the specimens as follows: (a) The chloride concentration profiles of the specimens in each case present a distinct decline and stable phase. (b) The chloride concentration in specimens is directly proportional to the immersion duration of the specimens. The concentration exhibits a rapid increase during the initial stage of immersion, followed by a gradual deceleration. (c) The chloride concentration in the specimens is inversely proportional to the chloride diffusion depth. The concentration declines with depth and stabilizes at a certain depth. (d) The chloride migration in the specimens can be regarded as non-stationary diffusion with regard to time and space. The above phenomenon is explained by the mechanism of chloride migration in concrete. The dynamics of chloride migration in water-saturated specimens are mainly provided by chloride concentration gradients between adjacent pores [34,35]. Concrete sprayed with NDPS resulted in a reduction in chloride migration channels in the concrete. However, the dynamics of chloride migration did not change.

Chloride penetration resistance of NDPS-sprayed concrete is significantly improved as shown in Figure 9. At the same immersion time, the diffusion depth and accumulation of chloride in the specimens sprayed with NDPS were significantly reduced compared with the untreated specimens. And it can be found that the protective effect of NDPS is influenced by *w*/*c* and the NDPS spray volume.

#### 3.3.1. Influence of NDPS Spray Volume

The influence laws of NDPS spray volume on the protective effect of NDPS were revealed by comparing the chloride concentration profiles for specimens with the same *w*/*c* in Figure 9. It is clearly evident that the chloride diffusion depth in concrete with the same *w*/*c* decreases with the increase in NDPS spray volume. Comparing the chloride concentration profiles of W06D0 and W06D1000, it is shown that the chloride diffusion depth decreased from 20–25 mm to 15–20 mm with the increase in NDPS spray volume from 0 mL/m^2^ to 1000 mL/m^2^ for the specimens with *w*/*c* of 0.6 after 150 d of immersion.

A comparison of chloride concentration at an average depth of 2.5 mm for specimens with the same *w*/*c* immersed for 150 d is shown in Table 5. The influence of the NDPS spray volume on the chloride concentration in the specimen is more significant. The influence law can be summarized as follows: (a) The chloride concentration in the specimen decreases as the NDPS spray volume increases. (b) The decreasing trend in specimens with *w*/*c* of 0.5 and 0.6 slows down as the NDPS spray volume increases. (c) The decreasing trend in specimens with *w*/*c* of 0.4 initially intensifies and subsequently slows down with increasing NDPS spray volume.

The above phenomenon is mainly caused by the degree of pore blockage in the concrete intensifying as the NDPS penetrates the concrete. Chloride is more difficult to migrate in concrete as the pores are gradually blocked [36]. However, the influence of NDPS on concrete is limited. Once the active substances in NDPS reach saturation within the pores of the concrete, further increasing the spray volume of NDPS fails to significantly enhance its protective efficacy.

#### 3.3.2. Influence of *w*/*c*

The influence regularity of *w*/*c* on the protective effect of NDPS was revealed by comparing the chloride concentration profiles for specimens with the same NDPS spray volume in Figure 9. It is evident that the chloride diffusion depth in concrete with the same NDPS spray volume increases with *w*/*c*. Comparison of the chloride concentration profiles of W06D1000 and W04D1000, it is shown that the chloride diffusion depth decreases from 15–20 mm to 10–15 mm with the reduction in *w*/*c* from 0.6 to 0.4.

A comparison of chloride concentration at an average depth of 2.5 mm for specimens with the same NDPS spray volume immersed for 150 d is shown in Table 6. The influence of the NDPS spray volume on the chloride concentration in the specimen is more significant. The influence law can be summarized as follows: (a) In general, the protective effect of NDPS weakens as the *w*/*c* decreases. (b) The degree of influence of *w*/*c* on the protective effect of NDPS with different spray volumes is in the following order: 300 mL/m^2^ group > 1000 mL/m^2^ group > 500 mL/m^2^ group. (c) The influence of *w*/*c* on the specimens sprayed with 500 mL/m^2^ of NDPS appears to be relatively insignificant.

The phenomenon is probably explained by the fact that the compactness of concrete improves with the reduction in *w*/*c*, thereby leading to more difficulty for the NDPS to penetrate within the concrete and function [37]. Furthermore, as the *w*/*c* of concrete decreases, the number of pores with specific diameters that NDPS acts upon diminishes [11], resulting in a reduction in the protective effectiveness of NDPS.

It is further observed from Figure 9 that the growth rate of chloride concentration in the shallow layer of specimens sprayed with NDPS is slower than that of the untreated specimens. The chloride concentration on the surface of the specimens sprayed with NDPS was far below that of the untreated specimens over the immersion time. This is caused by the effective substances in NDPS remaining active in the concrete for a long time and continuing to react in the aqueous environment. Thereby, the chloride penetration resistance of concrete is continuously improved [38].

Based on the influence law of spray volume and *w*/*c* on the protective effect of NDPS and the characteristics of NDPS, the following suggestions are proposed for the application of NDPS in practical engineering as follows: (a) Considering the economic factors, the NDPS spray volume can be reduced appropriately to protect the concrete with high *w*/*c*. (b) To ensure adequate protection for concrete with a low *w*/*c*, it is necessary to employ a correspondingly larger spraying volume of NDPS. (c) The recommended spray volume of NDPS provided by the manufacturer is deemed reasonable and suitably applicable to concrete with medium to high *w*/*c*, ensuring optimal protective performance. (d) Ensuring that the concrete substrate remains saturated with water enhances the protective effect of NDPS.

## 4. The Chloride Transport Prediction Model in Concrete Sprayed by NDPS

### 4.1. Basic Model and Parameter Analysis

#### 4.1.1. Fick’s Second Law in the Submarine Environment

The non-stationary diffusion process of chloride in concrete is usually described quantitatively by Fick’s second law, shown in Equation (2) [39]:

(2) $$\frac{\partial C}{\partial t}=\frac{\partial }{\partial x}(D\frac{\partial C}{\partial x})$$

If the diffusion of the chloride is one dimensional, its analytical solution is given by Equation (3):

(3) $$C(x,t)=C_{0}+(Cs-C_{0})\cdot [1-erf(\frac{x}{2\sqrt{D_{app}\cdot t}})]$$

where *C*(*x*, *t*) represents the chloride concentration at depth *x* from the surface of the concrete at immersion time *t* (s), *C*_0_ represents the initial chloride concentration of the concrete (%), *C_s_* represents the surface chloride concentration of the concrete (%), *D_app_* is the apparent chloride diffusion coefficient of the concrete (m^2^/s), and erf is the error function.

The model neglects the impact of material properties and immersion time on *C_s_* and *D_app_*, leading to limited accuracy and reliability. In addition, pore blockage by NDPS results to a diminished absorption capacity of the concrete, thereby, the existing models are not sufficient to accurately predict the diffusion of chloride within in. It is necessary to make adjustments based on actual conditions to improve its performance.

#### 4.1.2. Parameter Analysis

The form of chloride migration in the concrete sprayed with NDPS was unaltered, thus Equation (3) is applicable for the calculation of *C_s_* and *D_app_* of the specimens. The use of Equation (3) to describe chloride diffusion in concrete is based on the following fundamental assumptions: (a) The pores are uniformly distributed within the concrete. (b) Concrete is a semi-infinite medium. (c) The surface chloride concentration of concrete is constant. (d) *C*_0_ represents both the initial chloride concentration of the concrete and the chloride concentration at infinite depth in the concrete. (e) The chloride concentration at the steady state phase in Figure 9 is adopted as *C*_0_. The concentration was taken as 0.01% in the calculations, due to the minor discrepancies observed in the stable phase of the chloride concentration profiles.

**Figure 10:**
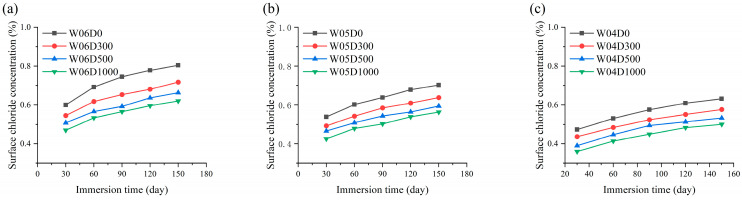
Surface chloride concentration with immersion time: (**a**) Cases with a *w*/*c* of 0.6; (**b**) Cases with a *w*/*c* of 0.5; (**c**) Cases with a *w*/*c* of 0.4.

The results of the calculation of the *C_s_* for specimens are shown in Figure 10. It can be concluded that the *C_s_* of each specimen exhibit a tendency to increase and then stabilize with the immersion time, and the growth rate is rapid at the initial stage of immersion. In addition, *C_s_* decreases with *w*/*c* and decreases with an increase in NDPS spray volume, and the influence of NDPS on *C_s_* diminishes gradually with *w*/*c*. In general, the rate of chloride entry into the concrete is slowed after being sprayed with NDPS. The above phenomenon is attributed to the more pores in concrete with higher *w*/*c*. Chloride migration is accelerated under this condition. However, the compactness of the concrete increases with the decrease in *w*/*c* and increases with NDPS spray volume. This leads to a more difficult diffusion and accumulation of chloride in concrete [40]. As a result, *C_s_* is smaller and slower to reach stability.

**Figure 11:**
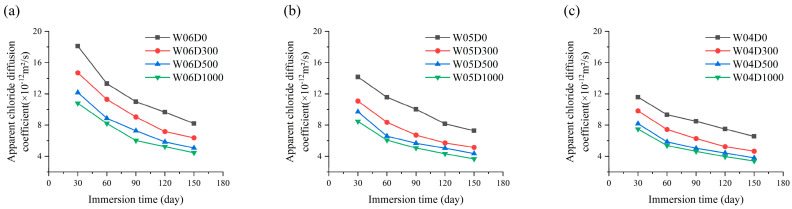
The apparent chloride diffusion coefficient with immersion time: (**a**) Cases with a *w*/*c* of 0.6; (**b**) Cases with a *w*/*c* of 0.5; (**c**) Cases with a *w*/*c* of 0.4.

The results of the calculation of the *D_app_* for specimens are shown in Figure 11. *D_app_* is commonly employed to evaluate the chloride penetration resistance of concrete. Chloride is more difficult to penetrate in concrete with lower *D_app_*. As shown in Figure 11, the *D_app_* of the specimens displayed a tendency to dwindle over the immersion time, and the decreasing rate of *D_app_* was larger in the early immersion. The chloride concentration gradually increases over the immersion time, and the chloride concentration gradient gradually decreases as well. This results in a gradual difficulty for outside chlorides to enter the concrete after a certain immersion time. The macroscopic presentation indicates a decrease in concrete *D_app_*. Moreover, as shown in Figure 11, the *D_app_* of concrete sprayed with NDPS is lower and decreases at a slower rate as compared to untreated concrete. This is explained by the fact that the concrete sprayed with NDPS is more compact and chloride is difficult to transport and achieve saturation in it. All the above phenomena indicate that spraying NDPS effectively improves the chloride penetration resistance of the concrete.

### 4.2. Model Establishment

One-dimensional chloride diffusion time-dependent prediction models for concrete sprayed with NDPS were established by updating Fick’s second law in conjunction with the variation laws of *C_s_* and *D_app_*.

#### 4.2.1. Surface Chloride Concentration C_s_

Relevant studies indicate that regression analysis of *C*_*s*_(*t*) can be performed by square root, linear, exponential, power, and logarithmic functions [41,42,43,44,45]. The variation law of *C_s_* over the immersion time shown in Figure 10 can be described by the power function as in Equation (4):

(4) $$C_{s}(t)=a\cdot t^{b}$$

where *a* and *b* are additional regression parameters.

**Figure 12:**
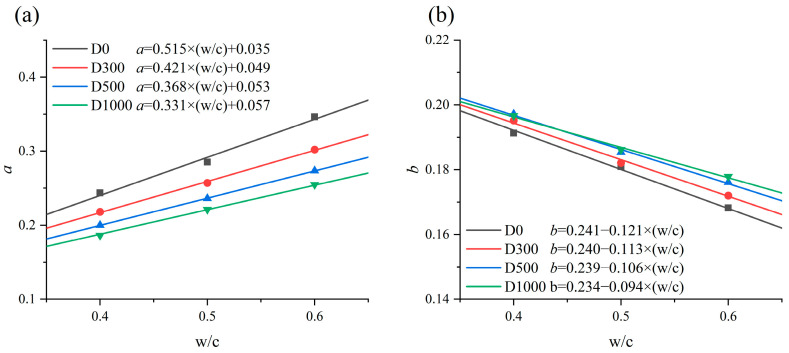
*a* and *b* with *w*/*c*: (**a**) Comparison of models and scatter for *a.* (**b**) Comparison of models and scatter for *b.*

The fitting results are shown in Table 7. This indicates that the regression parameters *a* and *b* are probably linearly related to *w*/*c* and the NDPS spray volume. The regression analysis proved that the regression parameters *a* and *b* are significantly correlated with *w*/*c*. Therefore, *a* and *b* are assumed to be the influencing factors of *w*/*c* in improving the accuracy of the model. This is specific to the model. Regression analyses were conducted by classifying the specimens into four cases, D0, D300, D500, and D1000, based on the NDPS spray volume. The results of the regression analysis are shown in Figure 12.

The empirical expressions for the variation in *C_s_* with the immersion time and *w*/*c* for D0, D300, D500, and D1000 obtained from the regression analysis using Equation (4) are shown in Equation (5):

(5) $$\left\{\begin{array}{l} C_{s,D0}(t,w/c)=[0.515\cdot (w/c)+0.035)\cdot t^{\left(0.241-0.121\cdot (w/c)\right)}]\\ C_{s,D300}(t,w/c)=[0.421\cdot (w/c)+0.049)\cdot t^{\left(0.240-0.113\cdot (w/c)\right)}]\\ C_{s,D500}(t,w/c)=[0.368\cdot (w/c)+0.053)\cdot t^{\left(0.239-0.106\cdot (w/c)\right)}]\\ C_{s,D1000}(t,w/c)=[0.331\cdot (w/c)+0.057)\cdot t^{\left(0.234-0.094\cdot (w/c)\right)}] \end{array}\right.$$

**Figure 13:**
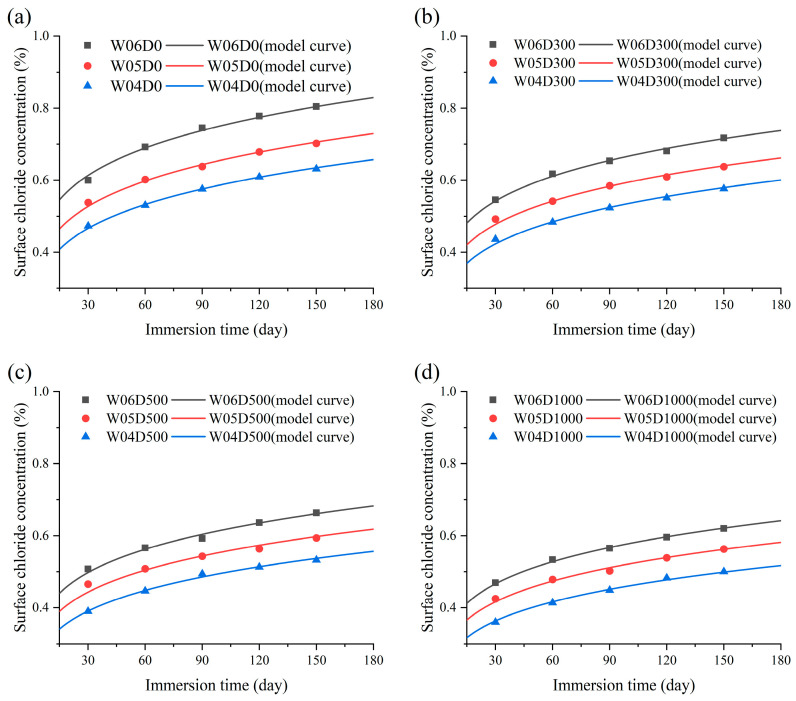
Comparison of the predicted *C_s_* by Equation (5) and *C_s_* fitted by Equation (3): (**a**) Case with NDPS spray volume of 0 mL/m^2^; (**b**) Case with NDPS spray volume of 300 mL/m^2^; (**c**) Case with NDPS spray volume of 500 mL/m^2^; (**d**) Case with NDPS spray volume of 1000 mL/m^2^.

The prediction results of Equation (5) are highly correlated with the fitting results of Equation (3), as shown in Figure 13. This supports the reasonableness of Equation (5).

#### 4.2.2. The Apparent Chloride Diffusion Coefficient *D_app_*

The *D_app_* are described usually by using the formula as shown in Equation (6) [46]:

(6) $$D_{app}(t)=D_{0}\cdot {\left(\frac{t_{0}}{t}\right)}^{n}$$

where *D*_0_ is the chloride diffusion coefficient (m/s^2^) of concrete at immersion time *t*_0_, *t* is the total immersion time, and *n* is the age attenuation coefficient. For calculation convenience, *t*_0_ is taken as the curing age of the specimen 28 d. At this time, Equation (6) becomes Equation (7):

(7) $$D_{app}(t)=D_{28}\cdot {\left(\frac{28}{28+t}\right)}^{n}$$

Table 8 presents the results of the regression analyses for *D_app_*. It is observed that *n* of the specimens increases with the NDPS spray volume and *w*/*c*. The law of *n* decreases with an increase in *w*/*c* is consistent with the relationship between *n* and *w*/*c* proposed by Mangat and Molloy [47]. The higher n in the NDPS-sprayed concrete than in the untreated concrete is probably explained by the optimization of the pore structure in the concrete resulting from the action of NDPS.

**Figure 14:**
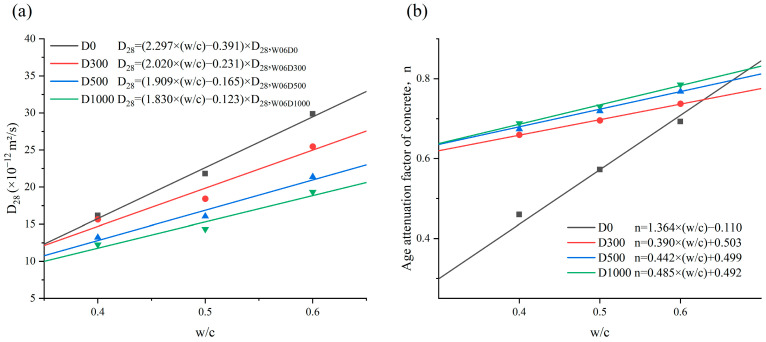
*D*_28_ and *n* with *w*/*c*: (**a**) comparison of models and scatter for *D*_28_; (**b**) comparison of models and scatter for *n*.

From Table 8, it is observed that *D*_28_ is influenced by the NDPS spray volume and *w*/*c*. The *D*_28_ of concrete decreases with the increase in NDPS spray volume and decreases with *w*/*c*. The protective effect of NDPS was evaluated in terms of *D*_28_. Regression analyses were conducted on *n* and *D*_28_ to improve the accuracy of the model and the results are shown in Figure 14. For example, the *D*_28_ of W06D1000 is approximately 35% lower than W06D0. The result is similar to that reported by Xiao et al. [21], and the reduction in the chloride diffusion coefficient observed is larger than the 20% reduction reported in the study by Ren et al. [48], which involved treating concrete with DPS. This demonstrates that spraying NDPS is an effective way to improve the chloride erosion resistance of concrete.

Combining Equation (7) and the results of regression analyses of *n* and *D*_28_, the empirical expression for the variation in *D*_app_ with immersion time and *w*/*c* at D0, D300, D500, and D1000 is obtained as shown in Equation (8):

(8) $$\begin{array}{l} D_{app}=D_{28}\cdot {\left(\frac{28}{28+t}\right)}^{n}\\ \left\{\begin{array}{l} D_{28,D0}(w/c)=[2.297\cdot (w/c)-0.391]\cdot D_{28,W06D0}\\ n_{28,D0}(w/c)=1.364\cdot (w/c)-0.110\\ D_{28,W06D0}=29.87\times 10^{-12}m^{2}/s \end{array}\right.\\ \left\{\begin{array}{l} D_{28,D300}(w/c)=[2.02\cdot (w/c)-0.231]\cdot D_{28,W06D300}\\ n_{28,D300}(w/c)=0.39\cdot (w/c)+0.503\\ D_{28,W06D300}=25.46\times 10^{-12}m^{2}/s \end{array}\right.\\ \left\{\begin{array}{l} D_{28,D500}(w/c)=[1.909\cdot (w/c)-0.165]\cdot D_{28,W06D500}\\ n_{28,D500}(w/c)=0.442\cdot (w/c)+0.499\\ D_{28,W06D500}=21.37\times 10^{-12}m^{2}/s \end{array}\right.\\ \left\{\begin{array}{l} D_{28,D1000}(w/c)=[1.83\cdot (w/c)-0.123]\cdot D_{28,W06D1000}\\ n_{28,D1000}(w/c)=0.485\cdot (w/c)+0.492\\ D_{28,W06D1000}=19.32\times 10^{-12}m^{2}/s \end{array}\right. \end{array}$$

**Figure 15:**
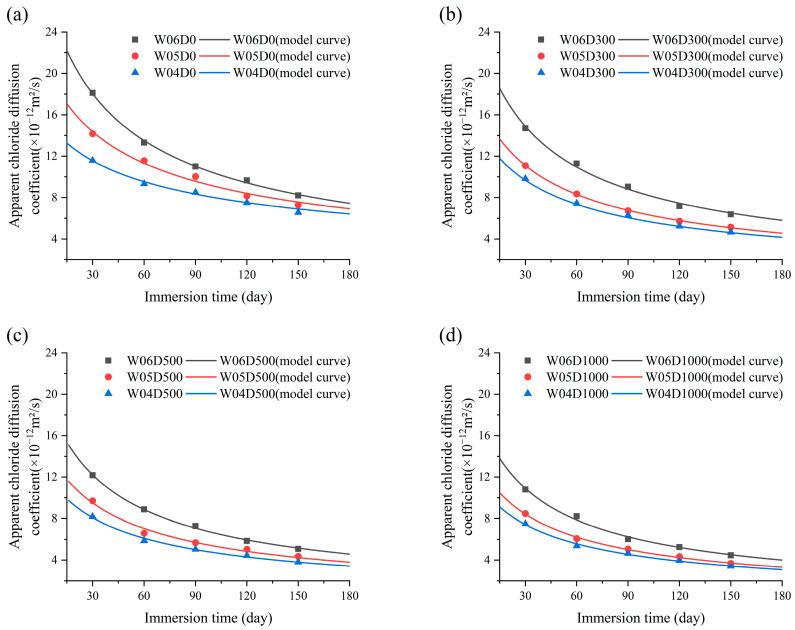
Comparison of the predicted *D_app_* by model and *D_app_* fitted by Equation (8): (**a**) Case with NDPS spray volume of 0 mL/m^2^; (**b**) Case with NDPS spray volume of 300 mL/m^2^; (**c**) Case with NDPS spray volume of 500 mL/m^2^; (**d**) Case with NDPS spray volume of 1000 mL/m^2^.

A comparison of the *D_app_* predicted by Equation (8) with the *D_app_* scatter fitted by Equation (3) is shown in Figure 15. It exhibits a good correlation, which supports the reasonableness of Equation (8). The one-dimensional chloride diffusion time-dependent prediction models for concrete sprayed with 0, 300, 500 and 1000 mL/m^2^ NDPS considering the influence of *w*/*c* were established by combining Equations (3), (5) and (8), respectively.

#### 4.2.3. Model Accuracy Validation

**Figure 16:**
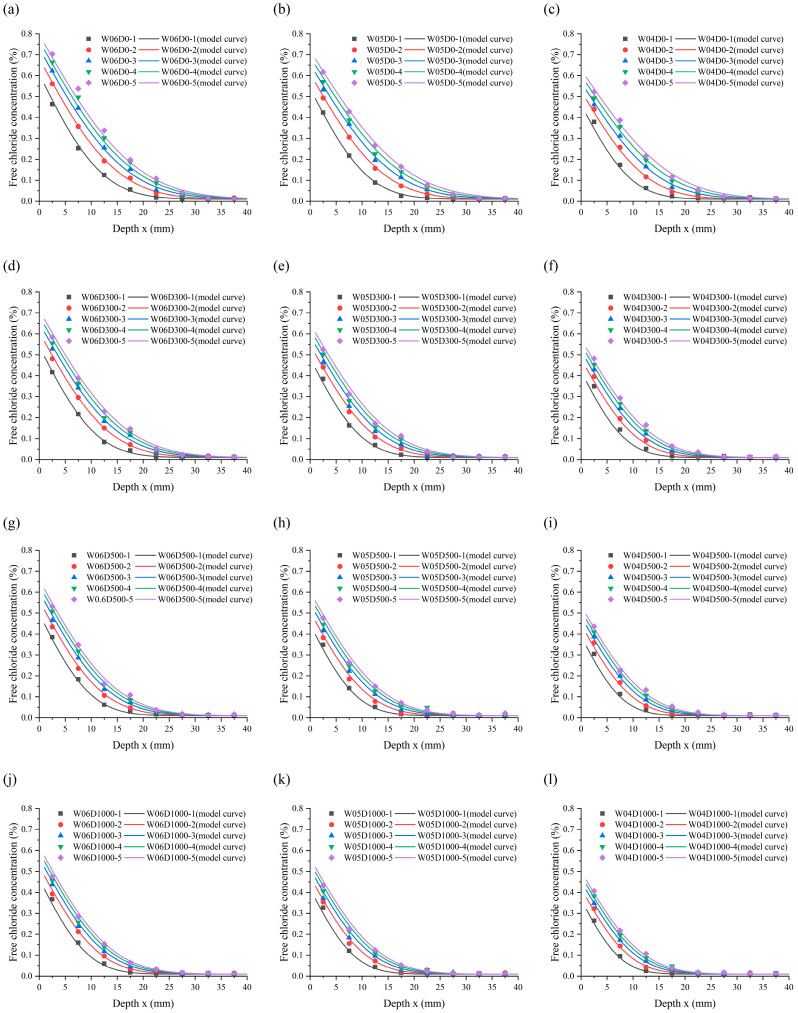
Comparison of the predicted chloride concentration by model and experimental chloride concentration: (**a**) Case W06D0; (**b**) Case W05D0; (**c**) Case W04D0; (**d**) Case W06D300; (**e**) Case W05D300; (**f**) Case W04D300; (**g**) Case W06D500; (**h**) Case W05D500; (**i**) Case W04D500; (**j**) Case W06D1000; (**k**) Case W05D1000; (**l**) Case W04D1000.

**Figure 17:**
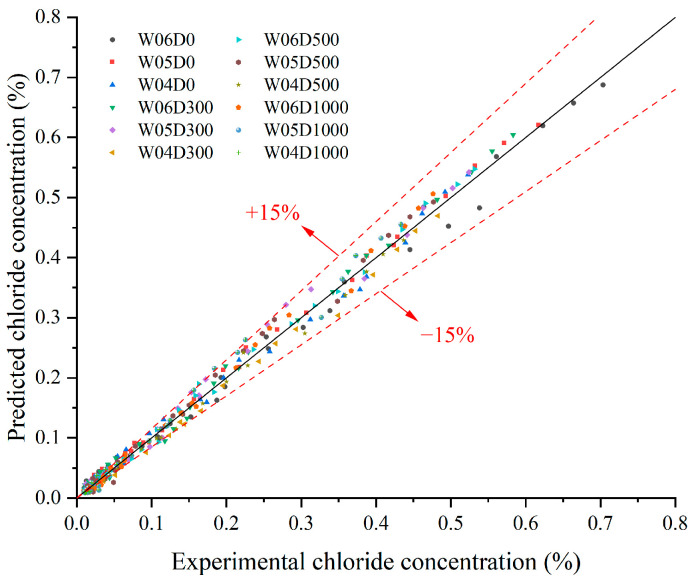
Corresponding relative error of the models.

Figure 16 presents a comparison of the predicted chloride concentrations by model with the experimental chloride concentrations. It is observed that the predicted chloride concentrations by the model closely matched the experimental chloride concentrations in the magnitude of the values and the trend of the profiles. The error analysis of the model-predicted and experimental values is shown in Figure 17. It can be observed that the percentage error rarely exceeds ±15%. This reflects a strong correlation between the model predictions and the experimental values. And the determination coefficient R^2^ is larger than 0.9. This demonstrates the superior applicability of the model.

**Figure 18:**
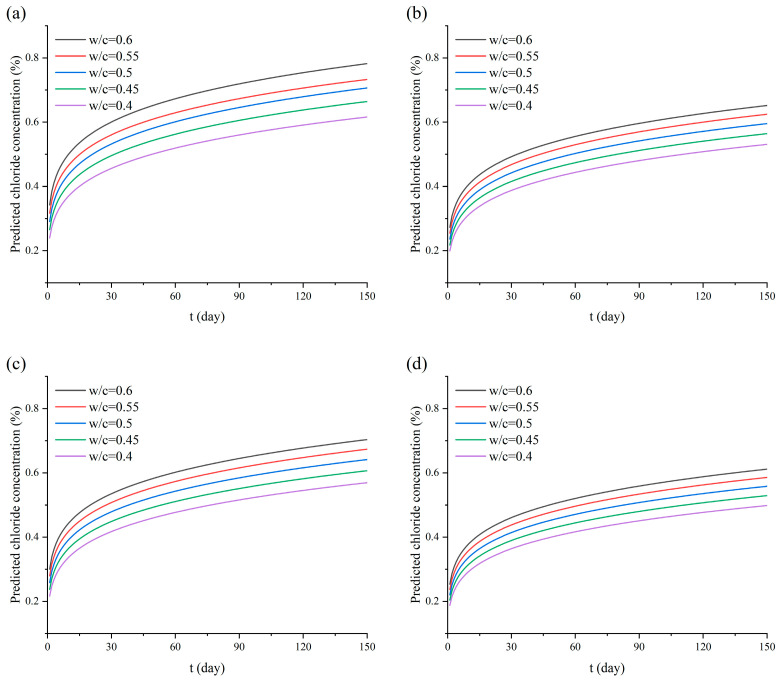
Model predictions for different *w*/*c*: (**a**) model for NDPS spray volume 0 mL/m^2^; (**b**) model for NDPS spray volume 300 mL/m^2^; (**c**) model for NDPS spray volume 500 mL/m^2^; (**d**) model for NDPS spray volume 1000 mL/m^2^.

Since the model primarily focuses on the influence of variations in the *w*/*c*, a sensitivity analysis was performed to determine the effect of the *w*/*c* on its predictive ability. The *w*/*c* of 0.4, 0.45, 0.5, 0.55, and 0.6 were selected to predict the chloride concentration variation in concrete at a depth of 2.5 mm over a 150-day period, the results are shown in Figure 18. It can be observed that the trend of the predicted chloride concentration variations is relatively consistent, which indicates that the model is more stable and less sensitive to variations in *w*/*c*.

In summary, the model is derived through rigorous refinement of Fick’s second law based on an indoor natural diffusion test. The model exhibits exceptional precision and stability, providing a basis for predicting the life cycle of concrete structures protected by NDPS for engineering practice. It is important to acknowledge that this model possesses inherent limitations. As a foundational model, it does not account for various environmental factors, including temperature, humidity, dry-wet cycling conditions, and seawater composition. Consequently, during practical applications, it may suffer from perturbations caused by these environmental variables, resulting in deviations from expected outcomes. Addressing these limitations represents a critical direction for future research endeavors.

## 5. Conclusions

In this study, the ability of NDPS to form an effective protective layer of sufficient thickness within concrete was verified. After that, the protective effect of NDPS to improve the chloride erosion resistance of concrete was specifically analyzed, and the influences of the NDPS spray volume and *w*/*c* on the protective effect were revealed. Finally, a prediction model was developed to predict the service life of concrete protected by NDPS. The following conclusions can be drawn:

(1) NDPS exhibits excellent permeation properties in concrete, forming a protective layer with a thickness similar to the concrete cover. The thickness of this protective layer varies based on two factors: it increases with the NDPS spray volume and decreases with *w*/*c*.
(2) NDPS possesses the ability to form an effective protective layer by blocking the pores within the concrete. The capillary water absorption coefficient of concrete dropped significantly after being sprayed with NDPS, and the decline rate of capillary water absorption coefficient increases with the NDPS spray volume and decreases with the increase in *w*/*c*.
(3) NDPS effectively improves the chloride resistance of concrete. The diffusion coefficient, diffusion depth, and concentration of chloride in concrete sprayed with NDPS are significantly lower than in unprotected concrete. The application of NDPS surface treatment can extend the service life of concrete structures exposed to chloride environments.
(4) The protective effect of NDPS is significantly influenced by the NDPS spray volume and *w*/*c*. At the same *w*/*c*, the protective effect of NDPS on concrete increases with the NDPS spray volume, and the growth rate gradually decreases. At the same NDPS spray volume, the protective effect of NDPS on concrete decreases with *w*/*c*, and the decline rate gradually increases.
(5) A one-dimensional chloride diffusion time-dependent prediction model considering the influence of *w*/*c* was developed based on Fick’s second law for concrete sprayed with NDPS. The predicted values of the model are in concordance with the experimental values with an error of not exceeding ±15%, which demonstrates the rationality and accuracy of the model. Although the model is developed based on indoor natural diffusion experiments, which inherently possess certain discrepancies compared to actual field conditions, it still provides a basis for predicting the life cycle of concrete structures protected by NDPS in practical engineering.

This study presents results obtained from short-term laboratory tests on concrete with medium to high *w/c,* thus there exists a certain disparity between these results and practical engineering applications. Based on this study, subsequent research will focus on enhancing the applicability of the model in practical engineering by incorporating environmental factors, such as temperature and wet–dry cycles.

## Figures and Tables

**Table 1:** The basic information of cement.

Cement Grade	Ingredients (%)					Setting Time (min)	
	SiO_2_	Al_2_O_3_	Fe_2_O_3_	CaO	MgO	Initial Setting Time	Final Setting Time
P.O. 42.5	24.99	8.26	4.03	51.41	3.71	172	234

**Table 2:** The mixture ratio of the concrete specimens (kg/m^3^).

*w*/*c*	Water	Cement	Sand	Crushed Stone
0.4	195	488	601	1116
0.5	195	390	635	1180
0.6	195	325	658	1222

**Table 3:** Test case design.

Group	Case Number	*w*/*c*	NDPS Spray Volume (mL/m^2^)
Control group	W04D0-t	0.4	0
	W05D0-t	0.5	0
	W06D0-t	0.6	0
Treatment group 1	W04D300-t	0.4	300
	W05D300-t	0.5	300
	W06D300-t	0.6	300
Treatment group 2	W04D500-t	0.4	500
	W05D500-t	0.5	500
	W06D500-t	0.6	500
Treatment group 3	W04D1000-t	0.4	1000
	W05D1000-t	0.5	1000
	W06D1000-t	0.6	1000

Note that in WxDy-t, x stands for the *w*/*c* of the specimen, y stands for the NDPS spray volume, and t stands for the immersion time (omitted for 0 day).

**Table 4:** Specimen groupings for EDS analysis.

Group	Case Number	Examination Factors
1	W04D0	Control group
	W05D0	
	W06D0	
2	W06D300	NDPS spray volume
	W06D500	
	W06D1000	
3	W04D300	*w*/*c*
	W05D300	
	W06D300	

**Table 5:** Comparison of chloride concentration in specimens with the same *w*/*c*.

Case Number	Reduction in Chloride Concentration (%)	Control Group Number
W06D300	17.08	W06D0
W06D500	24.40	
W06D1000	32.28	
W05D300	15.10	W05D0
W05D500	22.79	
W05D1000	29.85	
W04D300	7.75	W04D0
W04D500	16.50	
W04D1000	22.22	

Note that the chloride concentration represents the chloride concentration at 2.5 mm depth in specimens immersed for 150 d.

**Table 6:** Comparison of chloride concentration in specimens with the same NDPS spray volume.

Case Number	Reduction in Chloride Concentration (%)	Control Group Number
W06D300	17.08	W06D0
W05D300	15.10	W05D0
W04D300	7.75	W04D0
W06D500	8.82	W06D300
W05D500	9.06	W05D300
W04D500	9.50	W04D300
W06D1000	10.42	W06D500
W05D1000	9.14	W05D500
W04D1000	6.85	W04D500

Note that the chloride concentration represents the chloride concentration at 2.5 mm depth in specimens immersed for 150 d.

**Table 7:** Fitted parameters of surface chloride concentration.

Case Number	*a*	*b*
W04D0	0.346	0.168
W05D0	0.285	0.181
W06D0	0.244	0.191
W04D300	0.302	0.172
W05D300	0.257	0.182
W06D300	0.218	0.195
W04D500	0.274	0.176
W05D500	0.236	0.185
W06D500	0.2	0.197
W04D1000	0.254	0.178
W05D1000	0.221	0.186
W06D1000	0.186	0.197

**Table 8:** Fitted parameters of the apparent chloride diffusion coefficient.

Case Number	*D*_28_ (×10^−12^ m^2^/s)	*n*
W04D0	16.15	0.4596
W05D0	21.81	0.5724
W06D0	29.87	0.6927
W04D300	15.64	0.6594
W05D300	18.42	0.6956
W06D300	25.46	0.7373
W04D500	13.21	0.6736
W05D500	16.05	0.7189
W06D500	21.37	0.768
W04D1000	12.25	0.6881
W05D1000	14.34	0.7303
W06D1000	19.32	0.7851

## Data Availability

The original contributions presented in this study are included in the article. Further inquiries can be directed to the corresponding author.
